# A Solid Trap and Thermal Desorption System with Application to a Medical Electronic Nose

**DOI:** 10.3390/s8116885

**Published:** 2008-11-04

**Authors:** Xuntao Xu, Fengchun Tian, Simon X. Yang, Qi Li, Jia Yan, Jianwei Ma

**Affiliations:** 1 College of Communication Engineering, Chongqing University, Chongqing, 400044, P.R. China; 2 Mianyang Vocational and Technical College, MianYang, Sichuan, 621000, P.R. China; 3 School of Engineering, University of Guelph, Guelph, ON, Canada, N1G 2W1

**Keywords:** Medical electronic nose, solid trap/thermal desorption, detection limit, anti-disturbance

## Abstract

In this paper, a solid trap/thermal desorption-based odorant gas condensation system has been designed and implemented for measuring low concentration odorant gas. The technique was successfully applied to a medical electronic nose system. The developed system consists of a flow control unit, a temperature control unit and a sorbent tube. The theoretical analysis and experimental results indicate that gas condensation, together with the medical electronic nose system can significantly reduce the detection limit of the nose system and increase the system's ability to distinguish low concentration gas samples. In addition, the integrated system can remove the influence of background components and fluctuation of operational environment. Even with strong disturbances such as water vapour and ethanol gas, the developed system can classify the test samples accurately.

## 1. Introduction

An electronic nose is composed of a gas sensor array and a corresponding pattern recognition algorithm. It is able to imitate the olfaction system of humans and mammals, and to recognize odorant gases [1]. Currently, electronic noses have been applied to many areas such as quality control, environment monitoring [2], and disease diagnosis [3]. Human exhaled breath contains a large number of compounds (300-3,000 different compounds) [4]. Diseases can be diagnosed by utilizing the electronic nose to analyze volatile organic compounds (VOCs) in the exhaled breath of patients. As compared to traditional medical diagnostic methods such as blood and urine tests, the electronic nose is non-invasive, swift, convenient and efficient, thus attracting increasing attention from industrial and academic areas [5, 6]. The stable work concentration level of most gas sensors are in part per million (ppm) order, but the pathology marker gas concentration of human exhaled breath is between several parts per billion (ppbs) and several hundred ppbs [7]. A pre-condensation system is essential for the measurement for an enose to diagnose exhaled breath diseases under current precision levels of gas sensors.

## 2. Pre-condensation system

Common condensation methods for organic gases include solvent extraction, cryogenic trap, and solid trap condensation [8, 9]. Due to the dilution function of impregnation, the solvent extraction method cannot usually satisfy the requirements for ppb level analysis. Concentrating organic components using the cryogenic trap condensation method results in numerous concentrated vapors, which may incorrectly influence the analysis. The main difficulty with cryogenic sampling is the storage of the sample until analysis; therefore, almost all applications of the cryogenic collection of samples are followed by immediate analysis [10]. The solid trap condensation method has advantages such as easy control, low cost, high enrichment concentration efficiency, and the absence of organic and toxic impregnants; based on our literature survey, it is the best gas condensation method for medical electronic nose systems. In addition, the solid trap/thermal desorption gas condensation method has other advantages [11-13]: larger amounts of analytes can be concentrated on a solid sorbent out of a gas matrix through active or passive sampling; the sorbent packing and attendant equipment are small in size and light weight; the trap tube is in the relative front area of the gas path, which may reduce the manual disturbance; there are many choices of sorbents for different pathology marker gas, thus a good combination of different sorbents may allow for the determination of a wide range of VOCs; the humidity interference can be decreased by hydrophobic sorbents, dry gas purge, and splitting of the sample during analysis. With the advantages mentioned, the solid trap condensation method remains one of the most widely spread techniques for VOC analysis in practice. Despite its advantages, however, there are some bottlenecks that need to be addressed. First, variations in the split ratio affect the mass of analytics entering the detector relative to that loaded on the sorbent material. Second, the field samples taken either in environmental matrices, or in food, flavor, and fragrances typically contain up to hundreds of VOCs. Thus, the analyst has the difficulty of having a calibration mixture that contains all of the observed analytes.

**Figure 1.**
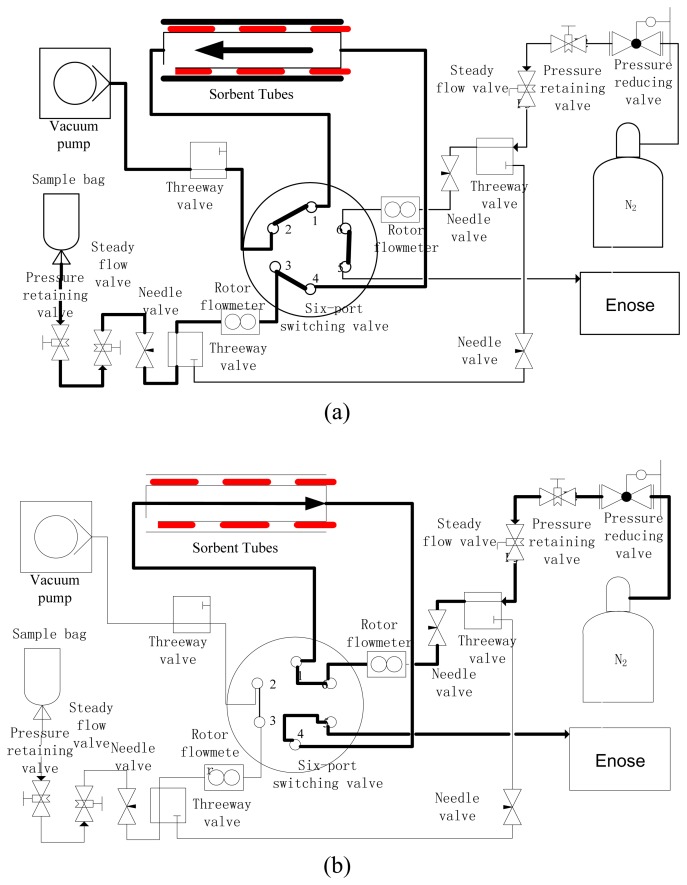
System layout of the pre-condensation for the e-nose. (a) Adsorption stage; (b) Desorption stage.

Figure 1 shows the system architecture of the pre-condensation system for the e-nose. At first, appropriate sorbents are selected for the object gas, and then the exhaled breath is transferred through the sorbents-filled trap tube. The VOCs of exhaled breath are then kept in the trap tube. The gas flow path of the trap stage is shown by the bold line in Figure 1(a). After sampling, the trap tube is heated to desorb VOCs. The object component then flows along with inert gas into the sensor chamber. We can utilize the different response patterns of exhaled breath to differentiate between patients and healthy people. The gas flow graph of the thermal desorption stage is shown by the bold line in Figure 1(b).

The solid trap/thermal desorption process can be treated as a dynamic quality balance process. The sample gas passes through the solid sorbents with constant speed, and the object component is trapped by the solid sorbents. The trapping process is usually composed of three stages: mass transfer, intragranular diffusion, and physical adsorption. The relationship between trapping capability and relative operation variables can be described as [14-16]:

(1) $$t_{b}=\frac{W_{e}W}{C_{0}Q}-\frac{W_{e}\rho_{B}}{k_{v}C_{0}}\ln\left[\frac{\left(C_{0}-C_{x}\right)}{C_{x}}\right]$$

where *t_b_* is the breakthrough time of the trap tube; *C_0_* is the gas concentration of sample from the inlet of the trap tube, g/cm^3^; *C_x_* is the gas concentration of sample from the outlet of the trap tube, g/cm^3^ ; *W* is the weight of sorbents, *W_e_* is the unit trapping capability of sorbents for special sample gas, g/g; *Q* is the volumetric flow of the sample gas, *ρ_B_* is the sorbents fill density, g/cm^3^; and *k_v_* is the trapping factor.

Clearly, the gas condensation capability of the solid trap/thermal desorption system is related to the following factors: the physical and chemical properties of the sorbent; the physical and chemical properties of the object gas; the initial concentration of the object gas; and experimental conditions such as temperature, gas flow rate, and relative humidity.

The selection of sorbents to be used is extremely important for the solid trap/thermal desorption technique. This choice is determined by four criteria [10]. First, a breakthrough of the analytes has to be avoided. Secondly, the sorbent should not produce any artifacts. The sorbent must be kept free of contamination before and after sampling. Finally, the retention of water with the sorbent material has to be as low as possible. According to the property and structure of the material, sorbents can be classified into inorganic adsorbents and organic porous polymer adsorbents. Active carbon, graphitized carbon black, and carbon molecular sieve are inorganic adsorbents, while Tenax®, Porapak™, Chromosorb®, and Amberlite™ are organic porous polymer adsorbents. Inorganic adsorbents have the advantages of a big surface and high work temperature, excellent trapping capability, high trapping quantity, and good thermal stability. They are usually used to enrich volatile and semi-volatile compounds. However, most inorganic adsorbents are hydrophilic to require high thermal desorption temperature with excess active points at the sorbent surface. Thus, inorganic adsorbents can easily cause incomplete desorption or irreversible decomposition. On the other hand, organic porous polymer adsorbents are commonly used with the advantage of relatively low desorption temperature. The sorbents for the concentrating system of the medical electronic nose are decided by simultaneity considering the marker VOC of disease and the pre-condensation technique at the same time.

**Figure 2.**
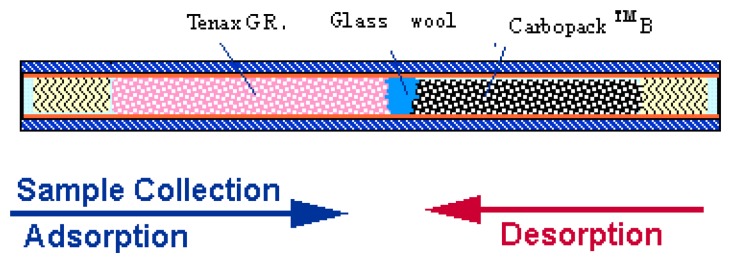
Multi-bed sorbent tube.

For example, breast cancer is accompanied by increased oxidative stress and the induction of polymorphic cytochrome P-450 mixed oxidase enzymes (CYP). Both processes affect the abundance of VOCs in the breath because oxidative stress causes lipid peroxidation of polyunsaturated fatty acids in membranes, producing alkanes and methylalkanes that are catabolized by CYP. In [17], the VOCs in 1.0 L of breath and 1.0 L of room air were captured onto separate sorbent traps, and samples were analyzed by gas chromatography and mass spectroscopy. The breath methylated alkane contour (BMAC) in each subject was constructed using the alveolar gradients of C_4_–C_20_ *n*-alkanes and monomethylated alkanes. BMACs in women with and without breast cancer were compared. According to their discriminatory power as markers of breast cancer, forward stepwise discriminant analysis identified eight VOCs in the BMAC as the best markers of breast cancer. The eight VOCs are nonane, 5-methyltridecane, 3-methylundecane, 6-methylpentadecane, 2-methylpropane, 3-methyl-nonadecane, 4-methyldodecane and 2-methyloctane. The breath test distinguished between women with breast cancer and healthy volunteers with a sensitivity of 94.1% (48/51) and a specificity of 73.8% (31/42) using these eight VOCs. Using five other breast cancer biomarker VOCs, the breath test distinguished between women with breast cancer and healthy volunteers with a sensitivity of 93.8% and a specificity of 84.6%. Those five VOCs are 2-propanol, 2,3-dihydro-1-phenyl-4(1*H*) quinazolinone, 1-phenylethanone, heptanal, and isopropyl myristate [18]. According to the trapping capability of gas, we choose the following sorbents: Carbopack™B and Tenax® GR, adopt OD1/4 inch, 7-inch length standard sorbent tube, and filling with 120-150 mm (about 600∼1,000 mg) mixed sorbents, which can complete the sampling of object gas. The multi-bed sorbent tube is shown in Figure 2. The tube consists of 60 mm Tenax® GR plus 60 mm of Carbopack™B separated by 3 mm of glass wool. These adsorbents are arranged in order of increasing sorbent strength from the sampling end of the tube. During the filling process, the sorbents should not be too tight to stress the sorbents or too loose to leave holes at the sorbent bed.

The system combines a pressure retaining valve, steady flow valve, needle valve and rotor flowmeter to precisely control the flow. The sample gas and pure nitrogen pass the pressure retaining valve first, which can guarantee that when the pressure of the sample and pure nitrogen changes, the pressure of the next steady flow valve would not change. The steady flow valve restrains the flow trend of gas resistance changes caused by temperature changes in the gas path. The flow is adjusted by a needle valve with a scale tray. To keep the system airproof, the system gas path adopts a 3 mm stainless steel pipe, and the system gas path is connected with pipes using ferrule-fitting connection.

According to the trapping theory, the cooler the system is, the stronger the trapping capability. When temperature increases, the trap capability between the sorbents and the trap objects decreases. The trapping efficiency of the adsorbent traps tends to increase exponentially with a decrease in temperature [19]. Thus, the faster the temperature increases and the higher the final temperature, the faster is the desorption speed. A low thermal desorption temperature may cause the sample components to desorb incompletely, while a high temperature may cause low recycle efficiency because of the instability of components. Obviously, the temperature of trapping and desorption may significantly influence gas condensation effects. To guarantee the repeatability of experiments, the system must achieve precise temperature control for the trapping and desorption process. According to the characteristics of the chosen sorbents and sample gas, a quartz lamp is adopted for heating, and a semiconductor peltier instrument is adopted for cooling. Table 1 shows the details of the heater and the cooler. The studies in [20, 21] indicate that the sorbent has better trapping efficiencies in a lower temperature, but because we used only a one-stage Peltier cooling system, we discovered that when the trapping temperature was lower than 5 °C, the cooler was influenced by environmental temperature obviously and affected the stability of temperature control in our experiments. For our subsequent research, we plan to decrease the trapping temperature by a two-stage Peltier cooling system or increasing the power of the cooler

## 3. The electronic nose

**Figure 3.**
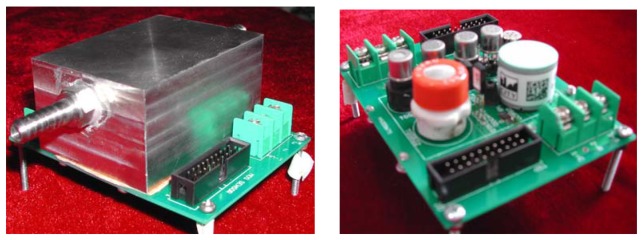
The electronic nose (left) and its sensor array (right).

To improve gas condensation effects, the chamber of the enose needs to be made as small as possible. Our electronic nose system adopts a special design method. In Figure 3, the sensor array is composed of six metal oxide sensors, an electric chemical sensor and temperature, humidity, and pressure sensors. The gas sensors are selected according to their sensitivity for the object gas, while the temperature, humidity, and pressure sensors can increase the capability for restraining the drift. The chamber of the electronic nose covers the sensor arrays directly, which utilizes a sealed gasket and screw to guarantee an airproof system. The available volume of the chamber is 72 mL. The signals of gas sensors and the temperature, humidity, and pressure sensors are transferred to signal regulating circuitry. The electric signal is sampled into a computer through a 14-bit data acquisition card (USB2002, Beijing ART-Control Inc.).

## 4. Result and Discussion

We conducted a series of laboratory experiments to validate the developed system. We confirmed the influence of the parameters such as temperature and flow rate on the condensation effect of the system by observing the change in the condensation effect by changing the temperature of adsorption, temperature of desorption, and flow rate of desorption in Section 4.1. We tested the lowest limits of detection in the system to five components of a 1L sample volume in Section 4.2. We confirmed that the system can increase the differentiating capability to low concentration samples by comparing the test effect of the system to three types of low concentration samples before and after condensation in Section 4.3. We confirmed the anti-interference capability of the system by comparing the test effect of the system to samples with high humidity and background interference before and after condensation in Section 4.4. We then repeated the same experiment three times in each group of experiments and analyzed the mean response value of the each repetition.

### 4.1. The influence of experiment parameters for system condensation effects

**Figure 4.**
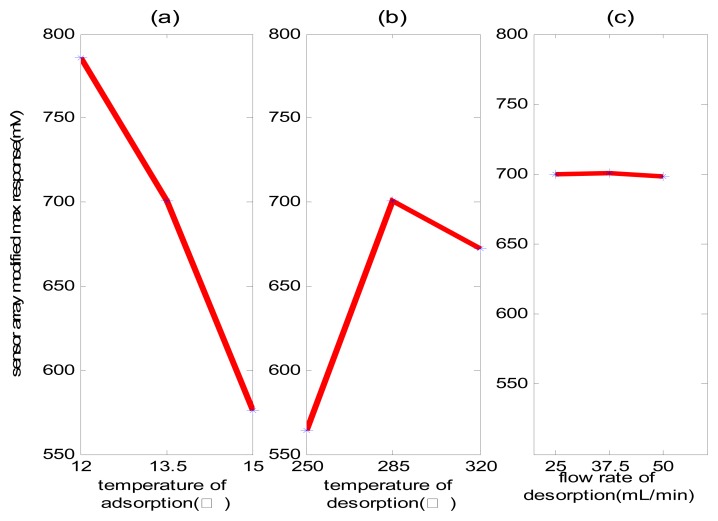
The influence of parameters for system condensation effects. (a) The influence of trapping temperature; (b) The influence of desorption temperature; and (c) The influence of desorption flow rate.

We used 50 ppb nonane to investigate the influence of different trapping temperatures, desorption temperatures, and desorption flow rate for system condensation effects. In all experiments, the sample volume was 500 mL. The condensation effects under different operating conditions (shown in Table 2) are shown in Figures 4(a)-(c). The y-axis is the average response of the sensor array, which indicates the concentration of concentrated gas.

In Figure 4(a), a lower trapping temperature results in better condensation effects. In Figure 4(b), the desorbing temperature cannot be too high or too low as a low temperature causes incomplete desorption and high temperature leads to the decomposition of object gas, which may influence the condensation effects. The seal heating desorption is introduced in Figure 4(c), showing that the influence of condensation effects on the desorption flow rate is reduced in selected flow rate ranges. According to [22], the flow rate of desorption cannot be set too low; otherwise, the efficiency of thermal desorption will be influenced. Different object components with different chemical properties, likewise have dissimilar the corresponding temperatures and flow speeds in optimal system condensation effects. For special application, the experiment design method can be adopted to determine the optimal system parameter.

### 4.2. Limit of detection decrease

A pre-condensation system is essential for the measurement to utilize the electronic nose to diagnose exhaled breath disease under current precision of gas sensors. Gas condensation together with the medical electronic nose system can significantly increase the detecting ability of the electronic nose and lower the bottom limits of detection. First, 1 L of different concentrations of breast cancer character gas are allowed to pass through the trapping/thermal desorption system, and then these are tested using electronic nose. The lowest detection limits of gas condensation together with the electronic nose for five breast cancer character gases are shown in Table 3. The trapping temperature is 12 °C, the trapping flow rate is 100 mL/min, the desorption flow rate is 50 mL/min, and the desorption temperature shown in Table 3 varies according to different boiling points. The recovery rate of the solid trap/thermal desorption system is influenced by a number of factors [23]. Under the premise that operating conditions are invariable and the sample volume does not exceed the breakthrough volume, it is reasonable to expect that an increase in gas sample volume may further decrease the lowest limit of detection.

### 4.3. Increase of the differentiating capability

The electronic nose can detect a low-concentration component whose volume concentration is bigger than the lowest limit of detection; however, the recognition effect is unacceptable. Gas condensation together with the medical electronic nose system can increase the differentiating capability significantly.

The essence of principal component analysis (PCA) is to represent the information that original variables contain through fewer mutually uncorrelated variables by dimension reduction. The gas sensor array consists of seven gas sensors in our electronic nose system, and the original feature is characterized by signal of seven dimensions that cannot be visually expressed directly. To explain that the system can increase the differentiating capability, we carried out a PCA on the original feature

**Figure 5.**
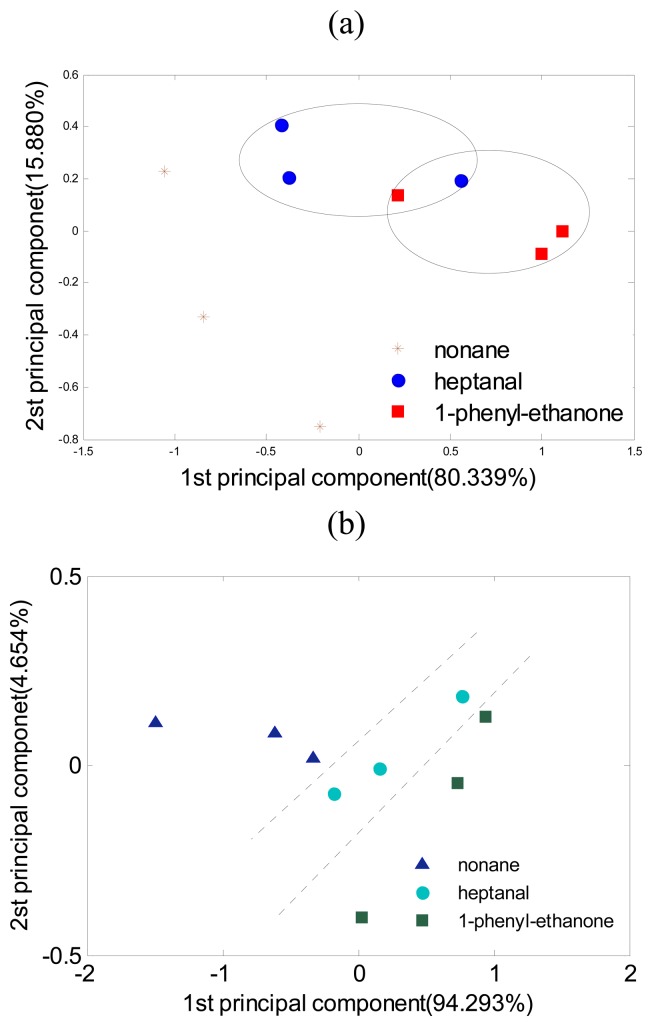
Principal component analysis of the measurement at three low concentration odorant gas samples. (a) Result using electronic nose alone, where the odorant gas components not is linear separable; (b) Result using gas condensation together with the enose, where the odorant gas components is linear separable.

In Figure 5, the object components are 60 ppb nonane, heptanal, and 1-phenylethanone. Figure 5(a) is the result of the tests that use electronic nose directly. Figure 5(b) shows the test result of gas condensation together with the electronic nose. In Figure 5(a), the low concentration means that the system differentiating capability is not enough, which leads to an overlap of sample heptanal and 1-phenyl-ethanone. After condensation, the system was able to classify the three samples correctly.

### 4.4. Gas condensation together with the electronic nose can improve the anti-interference capability

The electronic nose can detect a high concentration component directly with preferable results. However, when other factors such as humidity and temperature interfere with the test sample, the recognition result becomes unacceptable. Gas condensation together with the electronic nose can improve the anti-disturbance capability, and achieve preferable classifying capability.

Vapor interference is especially obvious in breath gas detection. To confirm the anti-vapor interference capability of the system, we adopt gas condensation together with the medical electronic nose system to test the samples with humidity interference.

**Figure 6.**
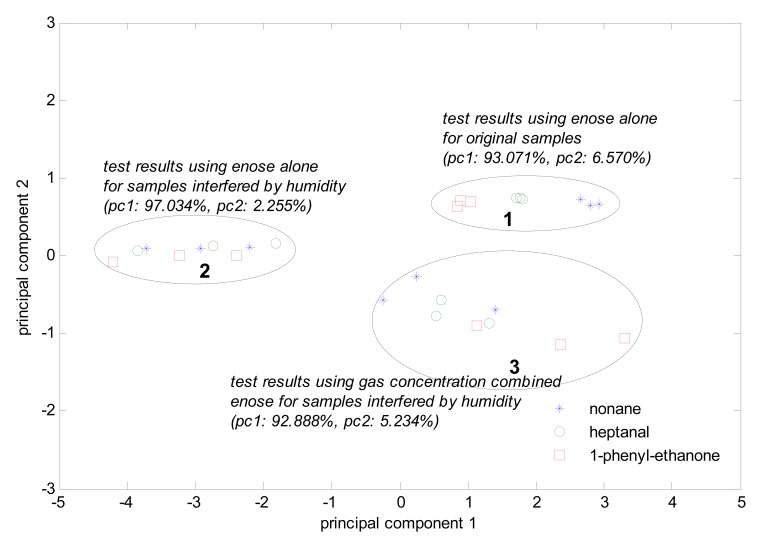
PCA of the three odorant gas samples strongly interfered by humidity.

In Figure 6, the object components are 200 ppb nonane, heptanal, and 1-phenylethanone. Set 1 shows the electronic nose test results for nine original samples. Set 2 shows the electronic nose test results for the samples with strongly humidity interference. Set 3 is the gas condensation together with the electronic nose test results for the samples strongly affected by humidity.

Obviously, the system has a preferable classifying capability for the nine original samples. However, when the samples are affected by strong humidity, the test results overlap and cannot be classified. When we use gas condensation together with the electronic nose, the humidity interference is reduced to the smallest possible size, and only two samples were be recognized inaccurately.

Aside from the interference factors of humidity and temperature, the medical electronic nose can dismiss interference from other background factors such as ethanol gas and other disinfectants, which may reduce system recognition capability.

**Figure 7.**
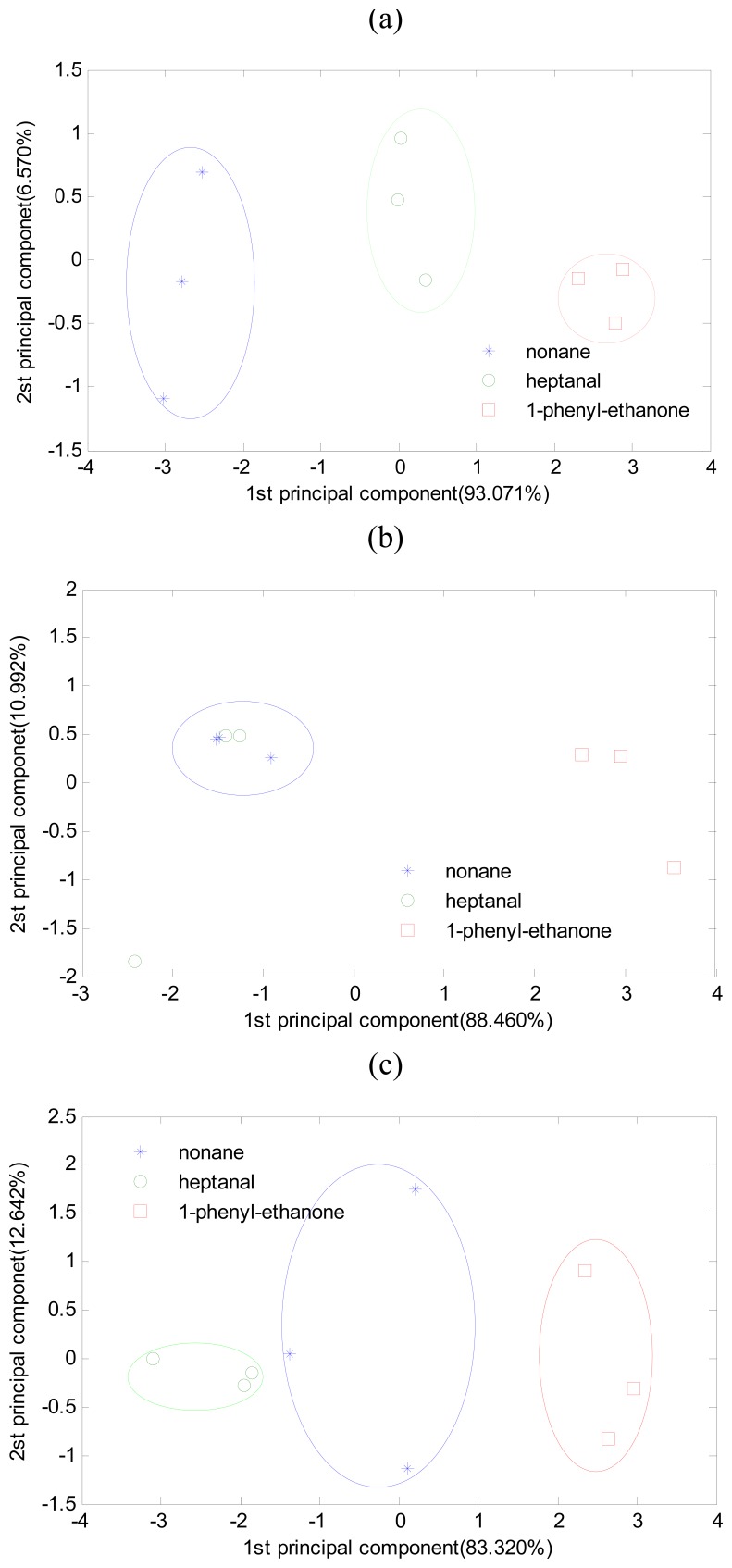
PCA of the three odorant gases sample strongly interfered by background factors. (a) Nine original samples can be classified correctly; (b) The interfered samples may cause overlap of nonane and heptanal samples; (c) The interfered samples can be classified correctly by gas condensation together with the electronic nose system.

By choosing appropriate sorbents, gas condensation together with the enose system can restrain high concentration background component interference and achieve effective recognition. In Figure 7 (a-c), the object components are 200 ppb nonane, heptanal, and 1-phenylethanone. The interference of 200 ppb ethanol gas meant that the test cannot be considered to classify correctly. However, all the gas samples were classified correctly when the system adopted gas condensation.

## 5. Conclusions

A solid trapping/thermal desorption-based gas condensation system is constructed in this research, which extends the concentration workspaces of current gas sensors. The key parameters of the developed system are discussed, and the theoretical analysis and experimental results indicate that:

1) The developed system has high precision in the operating parameters such as temperature and flow rate to guarantee the repeatability of the integrated system.
2) Choosing the appropriate sorbents can decrease the lowest limit of detection.
3) For samples whose volume concentration is higher than the lowest limit of detection, gas condensation and electronic nose system can increase the differentiation capability.
4) By choosing appropriate sorbents and introducing dry blow during the solid trapping/thermal desorption process, the developed system can improve anti-interference capabilities. The system has good restraining capability especially with humidity interference in breath gas detection.
5) When appropriate sorbents according to specific applications are chosen, the developed system can remove the interference of background components such as ethanol gas and other disinfectants, achieving effective recognition.

The final goal of the integrated system design is to use it in breast cancer detection according to exhaled breath. We have carried our research based on laboratory experiments. Our goal is to fully simulate an actual clinical environment and optimize the system performance following actual clinical detection, which will be our research emphasis in the next stage of our work.

## Figures and Tables

**Table 1.** The parameter of heater and cooler.

	**Heater**	**Cooler**
Temperature range (°C)	100-325	5-20
Precision (°C)	0.1	0.1
Thermal resistance	Pt.100	Cu.50
Controlling method	Fuzzy PID	Fuzzy PID
Power (W)	600	60
Volume of heater (mL)	2000	---

**Table 2.** The parameters in Figure 4.

	Figure 4(a)	Figure 4(b)	Figure 4(c)
Temperature of adsorption (°C)	12, 13.5, 15, respectively	13.5	13.5
Flow rate of adsorption (mL/min)	60	60	60
Temperature of desorption (°C)	285	250, 285, 320, respectively	285
Flow rate of desorption (mL/min)	37.5	37.5	25, 37.5, 50, respectively

**Table 3.** Limits of Detection (LOD) using the integrated pre-condensation and e-nose system. Sample volume is 1 L; trapping temperature is 12 °C, the trapping flow rate is 100 mL/min; desorption flow rate is 50 mL/min.

	**Compound**	**Temperature of desorption (°C)**	**LOD (ppb)**
1	Nonane	150	1
2	*iso*-Propyl alcohol	250	1.5
3	Heptanal	150	1.25
4	1-Phenylethanone	200	2
5	Isopropyl myristate	150	6
